# Delivery of Human EV71 Receptors by Adeno-Associated Virus Increases EV71 Infection-Induced Local Inflammation in Adult Mice

**DOI:** 10.1155/2014/878139

**Published:** 2014-08-27

**Authors:** Hung-Bo Hsiao, Ai-Hsiang Chou, Su-I Lin, Shu-Pei Lien, Chia-Chyi Liu, Pele Chong, Chih-Yeh Chen, Mi-Hua Tao, Shih-Jen Liu

**Affiliations:** ^1^National Institute of Infectious Diseases and Vaccinology, National Health Research Institutes, Zhunan, Miaoli 35053, Taiwan; ^2^Graduate Institute of Immunology, China Medical University, Taichung 40402, Taiwan; ^3^Institutes of Biomedical Sciences, Academia Sinica, Taipei 11529, Taiwan; ^4^Graduate Institute of Microbiology, National Taiwan University, Taipei 10051, Taiwan

## Abstract

Enterovirus71 (EV71) is now recognized as an emerging neurotropic virus in Asia and one major causative agent of hand-foot-mouth diseases (HFMD). However potential animal models for vaccine development are limited to young mice. In this study, we used an adeno-associated virus (AAV) vector to introduce the human EV71 receptors P-selectin glycoprotein ligand-1 (hPSGL1) or a scavenger receptor class-B member-2 (hSCARB2) into adult ICR mice to change their susceptibility to EV71 infection. Mice were administered AAV-hSCARB2 or AAV-hPSGL1 through intravenous and oral routes. After three weeks, expression of human SCARB2 and PSGL1 was detected in various organs. After infection with EV71, we found that the EV71 viral load in AAV-hSCARB2- or AAV-hPSGL1-transduced mice was higher than that of the control mice in both the brain and intestines. The presence of EV71 viral particles in tissues was confirmed using immunohistochemistry analysis. Moreover, inflammatory cytokines were induced in the brain and intestines of AAV-hSCARB2- or AAV-hPSGL1-transduced mice after EV71 infection but not in wild-type mice. However, neurological disease was not observed in these animals. Taken together, we successfully infected adult mice with live EV71 and induced local inflammation using an AAV delivery system.

## 1. Introduction

Hand-foot-mouth diseases (HFMD) are mainly caused by Coxsackie virus (CV) and enterovirus71 (EV71) infections and have become serious public health problems in Asia. In children, EV71 infections have been associated with fatalities and neurological complications [1–4]. Brain stem related insufficiency was the primary injury in patients with neurological impairment [5, 6]. The predominant pathological findings were in the thalamus, pons, midbrain, medulla oblongata, and spinal cord, with neutrophil and mononuclear cell infiltration. Neurogenic shock as a result of brain stem encephalitis has been proposed as the cause of pulmonary and cardiac complications [7]. Virus replication combined with damage to tissues with the induction of toxic inflammatory cytokines has been proposed as one possible pathogenesis [8–10]. Alteration of the cellular immunity of the host has also been suggested to be related to the severity of the disease [11–13]. In acute EV71 infections, massive IL-1*β*, IL-6, IFN*γ*, and TNF*α* secretion was observed in the serum and cerebrospinal fluid in patients with brainstem encephalitis and pulmonary edema (PE), which demonstrated a significant correlation between proinflammatory cytokines and the disease severity [8, 9, 14, 15]. Several EV71 candidate vaccines are presently being developed and evaluated in human clinical trials [16]. However, there is no cost-effective and rapid animal model that can be used to evaluate the potential protection effect of these vaccine candidates. The use of rhesus and cynomolgus monkeys as an infection model has been reported, but their use is limited for ethical and economic reasons [17, 18]. A mouse model has also been established, and one-day-old mice neonates were found to be susceptible to high infectious doses of EV71 [19]. There were also studies that used immunodeficient mice as the EV71-infection model. In 2008, Arita et al. established an EV71-infection model in NOD/SCID mice by using a mouse-adapted EV71 strain [EV71(NOD/SCID)], which induced paralysis of the hind limbs in 3- to 4-week-old NOD/SCID mice [20]. Recently, Khong et al. also demonstrated that 2-week-old and younger immunodeficient AG129 mice, which lack type I and II interferon receptors, are susceptible to infection with a non-mouse-adapted EV71 strain [21]. However, the immaturity and impairment of their immune system have greatly limited investigations, and, therefore, the data generated with neonate mice is debatable for regulatory organizations. Two receptors in humans for EV71 infection have been identified: P-selectin glycoprotein ligand-1 (PSGL1) and scavenger receptor class B, member 2 (SCARB2) [22, 23]. Recently, two reports have shown that human SCARB2 transgenic mouse could be infected by EV71 and that they exhibit a severe neurological disease, that is, similar to human encephalomyelitis in young mice [24, 25]. These results suggest that expression of the EV71 receptor in mice is a potentially feasible method for establishing an EV71 animal model.

Recombinant vectors based on adeno-associated virus (AAV) have been shown to stably express many genes* in vivo* without triggering immune responses to the vectors or transgenes. AAV vectors belong to the parvovirus family and are arguably the simplest vector, containing only a small, single-stranded DNA molecule encoding a protein, that is, flanked by inverted terminal repeats. To date, 11 serotypes and over 120 capsid variants have been categorized in six different phylogenetic clades representing the broad distribution of potential AAV biology [26]. The wide application of recombinant AAV vectors is because of their lack of pathogenicity, low immunogenicity, and most importantly, their ability to establish long-term transgene expression [27]. This technology is useful for systemic gene expression in animals to establish viral disease.

Although an EV71 receptor transgenic animal could be used as an EV71 animal model, it is not convenient to develop different strains or species of animals. Here, we report the successful generation of an animal model using an AAV vector that introduced PSGL1 and SCARB2 into an adult ICR mouse and changed their susceptibility to EV71 infection. Expression of human SCARB2 or PSGL1 in mice using the administration of recombinant AAV is reported in this study. After infection with EV71, the capsid protein VP1 of EV71 increased in the brain and intestines in both AAV-SCARB2- and AAV-PSGL1-transduced mice. Moreover, replication of EV71 was also found in receptors-transferred mice, inducing proinflammatory cytokines and chemokines in the tissues. It is well known that EV71 could not efficiently infect adult mice, so their immune system may play important roles to protect them from EV71 infection. The AAV gene transfer system is flexible and efficiently infects different strains of mouse for studying the immunopathogenesis of EV71 in adult mice. In the future, through the EV71 receptors gene-transfer into the innate receptor knockout mice the roles of innate immunity will be fully investigate.

## 2. Material and Methods

### 2.1. Construction and Expression of pAAV Vector Encoding EV71 Receptors

Plasmid pCMV-hSCARB2 containing the human SCARB2 gene was purchased from OriGene Technologies, Inc. (Rockville, MD). Plasmid pcDNA3-hmPSGL1 containing the extracellular domain (hECD) of human PSGL1 gene and transmembrane domain (TM) and cytosolic domain (CD) of mouse PSGL1 gene was purchased from Level technologies Inc. (Taiwan). The AAV plasmid pEMBL-CB-GFP containing the GFP transgene driven by the chicken *β*-actin promoter was described elsewhere [28]. To make the AAV plasmids, pEMBL-CB-hSCARB2 and pEMBL-CB-hPSGL1, the GFP sequence in pEMBL-CB-GFP was replaced, respectively, with the 1479 bp hSCARB2 and 1242 bp hPSGL1 sequences, which were obtained by PCR from plasmids pCMV-hSCARB2 and pcDNA3-hmPSGL1 using forward primer 5′-GTGAACCGGTGCCACCATGGGCCGATGCTGCTTC-3′ or 5′-ACCGGTATGCCTCTGCAACTCCTCC-3′ and reversed primer 5′-TCAAAGTTTACTGTCTCACGTTTTTAGCGTTAACCGTTCAATAGGGTGC-3′ or 5′-AGGGTTTAAACTAGGGAGGAAGCTGTGCA-3′, respectively. The inserted genes contain a flag gene for detection. The AAV vectors used in this study were from AAV serotype 8.

### 2.2. Mice

Specific pathogen-free ICR mice were purchased from the National Laboratory Animal Center of Taiwan. All of the mice used in this study were about 5~6 weeks old and the body weight of each mouse is around 15~20 g. Experiments were conducted according to the Laboratory Animal Center of the National Health Research Institutes (NHRI) of Taiwan guidelines. The animal use protocols have been reviewed and approved by the NHRI Institutional Animal Care and Use Committee.

### 2.3. Cells, Virus, and Antibodies

Human 293 cells were used to produce the recombinant AAV. L929 cells were used for the* in vitro* expression of pEMBL-CB-hPSGL1 and pEMBL-CB-hSCARB2, respectively. The non-mouse-adapted EV71 strain 4643-TW98 (GenBank: JN544418.1) used in this study was provided by Dr. J. R. Wang, National Cheng Kung University, Taiwan. This strain belongs to the subgenotype C2 and was originally isolated from throat swabs of an 18-month-old patient with encephalitis in Taiwan [29, 30]. Virus stocks were propagated in Vero cells using Dulbecco's modified Eagle's medium (DMEM; Gibco, MA, USA) supplemented with 10% fetal bovine serum (FBS). Monoclonal anti-EV71 antibodies (clone 422-8D-4C-4D, Millipore, MA, USA) were used to detect the EV71 viral protein [31, 32]; anti-human PSGL1 (BD Biosciences, CA, USA) and anti-human SCARB2 (Abcam, Cambridge, UK) antibodies were used to detect the expression of hPSGL1 or hSCARB2, respectively. Rabbit anti-EV71 antisera was generated in-house by using formalin-inactivated EV71 and used for immunohistochemical staining. Briefly, rabbits were immunized with inactivated EV71 three times at two-week interval. The sera were collected at the 8th week.

### 2.4. Flow Cytometry

pEMBL-CB-hPSGL1 and pEMBL-CB-hSCARB2 were transfected into L929 cells by using PolyJet* In Vitro* DNA Transfection Reagent (SignaGen Laboratories) according to user instruction. L929 cells transfected with pEMBL-CB-hPSGL1 were surface stained with anti-hPSGL1 antibody (BD Biosciences, 1 : 100 diluted) and followed with FITC-conjugated anti-mouse IgG (eBioscience, CA, USA; 1 *μ*g/mL). L929 cells transfected with pEMBL-CB-hSCARB2 were surface stained with anti-hSCARB2 antibody (Abcam, 1 : 100 diluted) and followed with PE-conjugate anti-mouse IgG (eBioscience, CA, USA; 1 *μ*g/mL). FACScaliber (BD Biosciences, CA, USA) was used for flow cytometry event collection and data were analyzed by CellQuest Pro software (BD Biosciences, CA, USA).

### 2.5. Western Blot

L929 cells were transfected with either pEMBL-CB-hPSGL1, pEMBL-CB-hSCARB2, or vector only, by using PolyJet* In Vitro* DNA Transfection Reagent (SignaGen Laboratories, MD, USA) according to user instruction. Followed with EV71 infection (MOI = 1.0), EV71 VP1 protein was detected by Western blot. Cell lysates were collected 48 hours after infection, and equal amount of total protein (50 *μ*g) from each sample was loaded into 10% acrylamide gel for electrophoresis, and then transferred to nitrocellulose (NC) membrane. The membrane was blotted with anti-EV71 antibodies (Millipore, 1 : 500 diluted) and secondary antibody (Zymed, CA, USA; 1 : 5000 diluted).

### 2.6. Production of Recombinant AAV

Human 293 cells were used to produce the recombinant AAV (rAAV-GFP, rAAV-hSCARB2, and rAAV-hPSGL1) by cotransfection with the pHelper and pXX8 plasmids. All of the rAAVs were produced by the triple transfection method as previously described and purified by cesium chloride sedimentation [33]. The physical vector titers were assessed by quantitative PCR [28] and the rAAV dosage used in this study defined as vector genome copies per milliliter (vg/mL).

### 2.7. Delivery Recombinant AAV into Mice

Different routes were used to deliver recombinant AAV-GFP into adult ICR mice. For intravenous (i.v.) route, each mouse was injected with recombinant AAV-GFP (5 × 10^12^ vg/mL) in 100 *μ*L PBS via tail vein. For intraperitoneal (i.p.) route, each mouse was directly injected 100 *μ*L of recombinant AAV-GFP (5 × 10^12^ vg/mL in PBS) into peritoneal. For intranasal (i.n.) route, each mouse was administrated with 100 *μ*L of recombinant AAV-GFP (5 × 10^12^ vg/mL in PBS) into nostrils. For oral route, each mouse was administrated with 100 *μ*L of recombinant AAV-GFP (5 × 10^12^ vg/mL in PBS) via gastric duodenal levin tube. Recombinant AAV-hSCARB2 and AAV-hPSGL1 (5 × 10^12^ vg/mL in PBS) were administrated in combination with 100 *μ*L for i.v. route and 100 *μ*L for oral routes. Adult ICR mice delivered with PBS in the same routes were used as control group.

### 2.8. EV71 Infection and EV71 Virus Titer Detection

Adult ICR mice were infected with 1 × 10^7^ pfu of EV71 (strain 4643) via i.p. injection in total volume of 100 *μ*L. Euthanized animals were perfused systemically with 50 mL of sterile PBS prior to organ harvesting. The tissue samples were homogenized in sterile PBS, disrupted by three freeze-thaw cycles, and centrifuged. The virus titers in the supernatants of the clarified homogenates were determined by plaque assays and expressed as plaque-forming units per milligram (pfu/mg). The plaque assay was performed by seeding Rhabdomyosarcoma (RD) cells in individual wells of a 24-well tissue culture plate and then infected with 10-fold serially diluted EV71 solution. Followed by one hour incubation at 37°C, 1 mL of 2.0% methyl cellulose in DMEM was overlaid onto the cells. The cultures were incubated at 37°C with a headspace of 5% CO_2_ for three days. The development of plaques was detected by removing the supernatant, washing the cells, and fixing them with 0.5 mL of 4.0% formaldehyde (BS Chemical, Taiwan). Monoclonal antibodies against EV71 viral protein (1 : 5000) were then added to the individual test wells. Following secondary antibody (anti-mouse IgG-HRP conjugated antiserum (Jackson ImmunoResearch, PA, USA at 1 : 5000 dilution) incubation and color development with TMB substrate (KPL), the numbers of viral plaques in each well were calculated [34].

### 2.9. Immunohistochemical Staining

Paraffin sections of mouse tissues were incubated with the rabbit anti-EV71 antisera (1 : 100), anti-hSCARB2 (1 : 200), or anti-hPSGL1 (1 : 200) at 4°C overnight. The sections were then washed three times with PBST and incubated with HRP-conjugated goat anti-rabbit IgG (1 : 200) or rabbit anti-mouse IgG (1 : 200) at 37°C for one hour. The sections were then developed with 3-amino-9-ethylcarbazole (AEC) and observed with a light microscope (Nikon ECLIPSE E600, Japan).

### 2.10. Real-Time RT-PCR

Total RNA was extracted from various organs of ICR mice by using Trizol reagent (Invitrogen, CA, USA) and purified by chloroform (Sigma-Aldrich, MO, USA) and isopropyl alcohol (Macron Chemicals, PA. USA). RNA (0.5–1 *μ*g) was reverse-transcribed to cDNA using an oligo-dT primer in a 20 *μ*L volume and SuperScript III RT (Invitrogen, Carlsbad, CA, USA) [35]. The mouse Universal Probe Library (UPL) set (Roche, Mannheim, Germany) was used to perform the real-time RT-PCR assay to measure gene expression. The specific primers for various genes and UPL numbers are listed in the Supplementary Material (available online at http://dx.doi.org/10.1155/2014/878139) (Supplemental Table 1). The related gene expression was calculated using the comparative method for the relative quantity normalized to GAPDH gene expression.

### 2.11. Statistical Analysis

A two-tailed *t*-test was used to analyze the statistical significance between individual groups. The results were considered statistically significance at *P* values of <0.05. The symbol “*” indicates a *P* value < 0.05, “**” indicates a *P* value < 0.01, and “***” indicates a *P* value < 0.001.

## 3. Results

### 3.1. Expression of Human SCARB2 and PSGL1 Increases EV71 Infection

**Figure 1:**
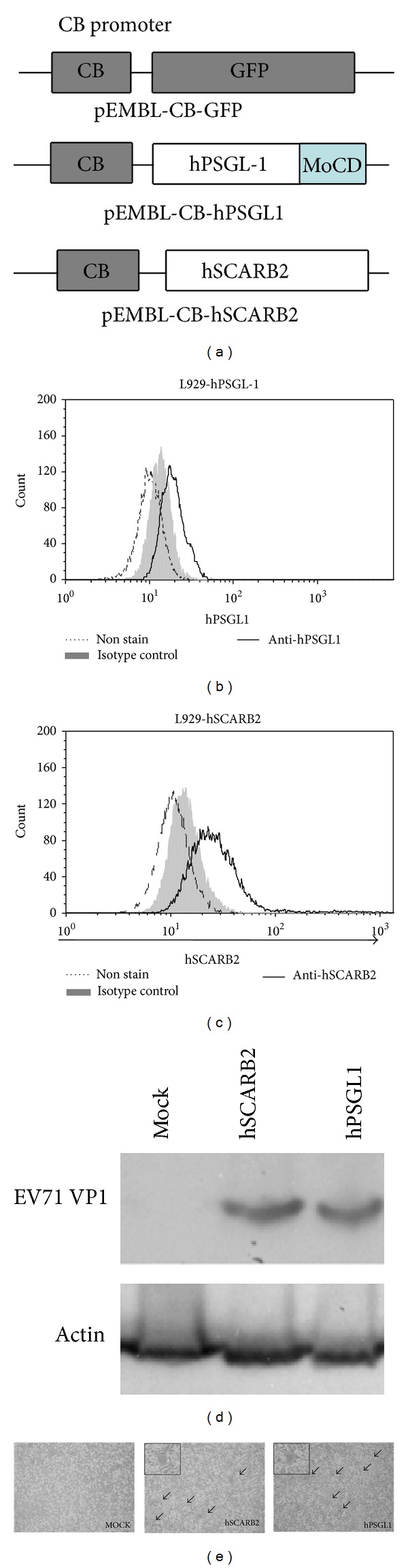
Expression of recombinant AAV-hSCARB2 and AAV-hPSGL1 in cell lines increased susceptibility to EV71 infection. (a) Green fluorescence protein (GFP), human PSGL1 (hPSGL1), and human SCARB2 (hSCARB2) were cloned into the pEMBL plasmid with a CB promoter (pEMBL-CB). (b) L929 cells were transfected with pEMBL-CB-hPSGL1 and the expression of hPSGL1 was detected by surface stained with anti-hPSGL1 antibody and FITC-conjugate anti-mouse IgG and then analyzed by flow cytometry. (c) L929 cells were transfected with pEMBL-CB-hSCARB2 and the expression of hSCARB2 was detected by surface stained with anti-human SCARB2 and FITC-conjugate anti-mouse IgG and then analyzed by flow cytometry. (d) L929 cells transfected with the vector pEMBL-CB (Mock), pEMBL-CB-hSCARB2 (hSCARB2), and pEMBL-CB-hPSGL1 (hPSGL1) were infected with live EV71 (MOI = 1.0). Forty-eight hours after infection, protein extracted from cells was analyzed by Western blot using anti-EV71 VP1 and anti-actin antibodies. (e) Identical L929 cell treatment as described in (d) was observed for the cytopathic effect (CPE) by microscopy. Cells with CPE are labeled with a black arrow.

The hSCARB2 and hPSGL1 genes were subcloned into the AAV vector pEMBL-CB-GFP to generate pEMBL-CB-hSCARB2 or pEMBL-CB-hPSGL1 (Figure 1(a)). In order to characterize the function of these plasmids, L929 cells were transfected with pEMBL-CB-hPSGL1 (Figure 1(b)) or pEMBL-CB-hSCARB2 (Figure 1(c)), and then the surface expression of hPSGL1 or hSCARB2 were analyzed by using flow cytometry. The results showed that L929 cells transduced with both plasmids did induced surface expression of hPSGL1 and hSCARB2. To further determine whether expression of hSCARB2 or hPSGL1 in mouse cells could increase cell susceptibility to EV71 infection, L929 cells were transfected with a mock control, pEMBL-CB-hSCARB2 or pEMBL-CB-hPSGL1. After 24 hours at 37°C, cells were infected with EV71 (MOI = 1.0) for another 48 hours. The expression of the EV71 capsid protein VP1 in EV71-infected cells was detected by Western blotting. Compared with the mock control, the VP1 protein levels increased in L929 cells expressing both hSCARB2 and hPSGL1 (Figure 1(d)). In addition, cytopathic effect (CPE) was observed in L929 cells expressing hSCARB2 or hPSGL1 which were infected with EV71, but not in the mock control (Figure 1(e)). By using immunofluorescence assay, we also confirmed that EV71 was detected in hSCARB2- and hPSGL1-expressed L929 cells, but not in the control cells (supplementary Figure S1). These results suggested that the expression of hSCARB2 or hPSGL1 facilitates the infection with EV71.

### 3.2. Determining the Delivery Route in ICR Mice Using rAAV-GFP

**Figure 2:**
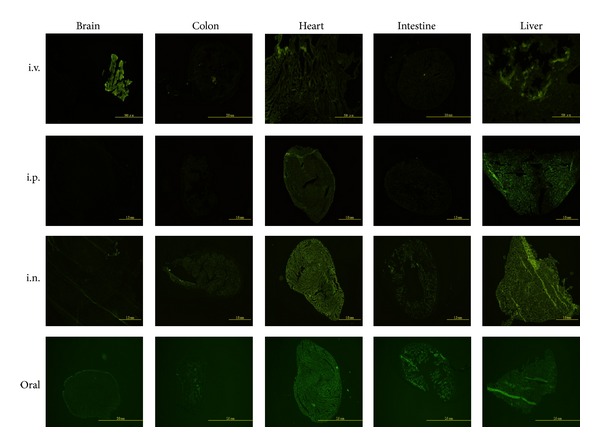
Delivery of rAAV-GFP by different routes induced diverse expression in different organs in the mice. Adult ICR mice were administered rAAV-GFP through different routes. Each ICR mouse was intravenously (i.v.) or intraperitoneally (i.p.) injected or intranasally (i.n.) or orally administered with 5 × 10^11^ vg of recombinant AAV-GFP. After three weeks, the mice were sacrificed and the organs, including the brain, colon, heart, intestines, and liver, were collected and frozen in sections. The expression of green fluorescence protein in the different organs was detected using a fluorescence microscope (OLYMPUS IX71, DP70, TH4-100). The data presented represent one of the three mice.

To confirm the expression levels of the rAAV transduced genes in different organs, we needed to determine suitable routes to deliver rAAV in mice. To this end, we generated rAAV-GFP, which expresses a green fluorescence protein after being delivered to an animal. The rAAV-GFP was administered to the ICR mouse by different routes and the green fluorescence that was expressed in different organs was analyzed at 3 weeks after administration. By using intravenous (i.v.) injection, strong green fluorescence occurred in the brain, heart, and liver (Figure 2). However, the majority of the green fluorescence was detected in the heart and liver by intraperitoneal (i.p.) or intranasal (i.n.) administration of rAAV-GFP (Figure 2). To increase the expression of green fluorescence in the intestines, rAAV-GFP was orally administered. Figure 2 shows that strong fluorescence was detected in the heart, intestines, and liver. These results indicated that different administration routes may have different distributions of AAV. To increase the viral infectivity in the intestines and brain, we decided to combine both intravenous and oral administration for delivering rAAV-hPSGL1 and rAAV-hSCARB2.

### 3.3. hSCARB2 and hPSGL1 Expression in Mice Increases EV71 Infection

**Figure 3:**
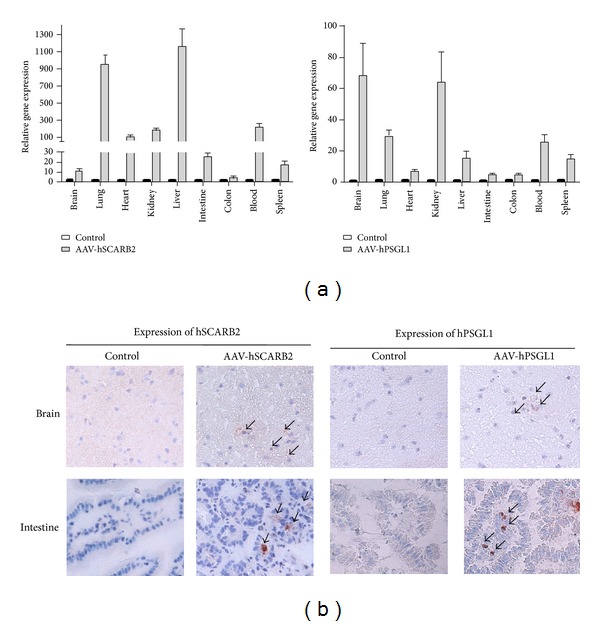
Expression of hSCARB2 and hPSGL1 in mice after rAAV delivery. ICR mice were administered 5 × 10^11^ vg of either rAAV-hSCARB2 or rAAV-hPSGL1 by the i.v. and oral routes. After three weeks, the mice were sacrificed and the organs, including the brain, heart, lung, spleen, intestine, colon, kidney, blood, and liver, were collected; RNA was extracted for real-time RT-PCR analysis (a). All of the data shown represent one of the three mice. Same organs were also collected from untreated ICR mice as control. The brain and intestines were also collected for frozen section and immunohistochemistry analysis. Expression of hSCARB2 and hPSGL1 was detected using the anti-hSCARB2 and anti-hPSGL1 antibodies, respectively (b). Positive cells are labeled with a black arrow.

Adult ICR mice were administered either 5 × 10^11^ vg of rAAV-hSCARB2 or rAAV-hPSGL1 by intravenous and oral administration and mice were sacrificed 3 weeks after administration to detect gene expression. Mice organs were collected, and hSCARB2 and hPSGL1 gene expression was determined by real time RT-PCR analysis.  Figure 3(a) shows that hSCARB2 and hPSGL1 could be detected in the brain, heart, lung, spleen, intestine, colon, kidney, liver, and blood after being delivered with rAAV-hSCARB2 and rAAV-hPSGL1, respectively. To further confirm hSCARB2 and hPSGL1 expression in the intestines and brain, tissue sections were stained with anti-SCARB2 or anti-PSGL1 antibodies and analyzed using immunohistochemistry (IHC) analysis (Figure 3(b)). The data show that both hSCARB2 and hPSGL1 could be expressed in adult mice after using the AAV delivery system.

**Figure 4:**
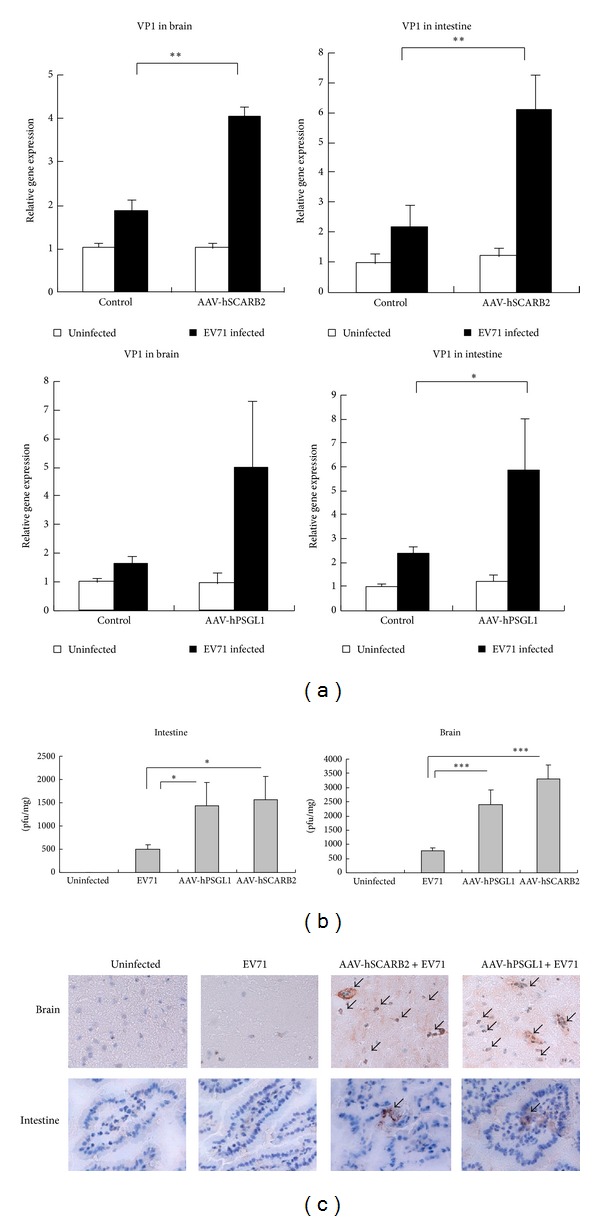
EV71 increased in the brain and intestines in mice transduced with rAAV-hSCARB2 and rAAV-hPSGL1. Adult ICR mice received 100 *μ*L AAV through i.v. and oral administrations of 5 × 10^12^ vg/mL of rAAV-hSCARB2 or rAAV-hPSGL1. After three weeks, the mice were or were not infected with the EV71 strain 4643 in 100 *μ*L (1 × 10^7^ pfu/mice) via an intraperitoneal (IP) injection. (a) Seventy-two hours after infection, RNA was extracted from brain- and intestinal-tissues. The transcript of EV71 VP1 was detected by real-time RT-PCR. *P < 0.05, **P < 0.01, and ***P < 0.001 (*t*-test). (b) Seventy-two hours after infection, brain and intestinal tissues were homogenized in PBS. The virus titers in the supernatants of the clarified homogenates were determined by a plaque assay and expressed as plaque-forming unit per milligram (pfu/mg) tissue. *P < 0.05, ^  ∗∗^ *P* < 0.01, and ^  ∗∗∗^ *P* < 0.001 (*t*-test). (c) Frozen sections of the brain and intestines were incubated with rabbit antiserum against EV71, followed by HRP-conjugated secondary antibody. Cells that stained positive are labeled with a black arrow. The results represent one of the three mice for each group.

To investigate whether the expression of EV71 receptors in mice increases EV71 infection, the AAV-transduced mice were i.p. infected with the non-mouse adaptive EV71 strain 4643 in 100 *μ*L (1 × 10^7^ pfu/mouse). After infection, there was no obviously abnormal behavior observed in the EV71 receptor-expressing mice or in the control mice in the 72 hours following infection. In contrast, EV71 VP1 transcripts were detected in the brain and intestinal tissues in EV71 receptor-expressing mice by real-time RT-PCR but were not detected in the control mice (Figure 4(a)). In addition, the mice tissues were collected for detecting live EV71 virus using a plaque assay. We found that the EV71 receptor-expressing mice had increased viral titers in the brain and intestines (Figure 4(b)). Furthermore, the tissue sections of the brain and intestines were stained with anti-VP1 antibodies by IHC analysis. These data indicate that VP1 was detected in the EV71 receptor-expressing mice but not in the control mice (Figure 4(c)). The results demonstrated that both rAAV-hSCARB2 and rAAV-hPSGL1-transduced mice are more susceptible to EV71 infection; however, infection did not induce neurological diseases.

### 3.4. Induction of Proinflammatory Cytokines and Chemokines

**Figure 5:**
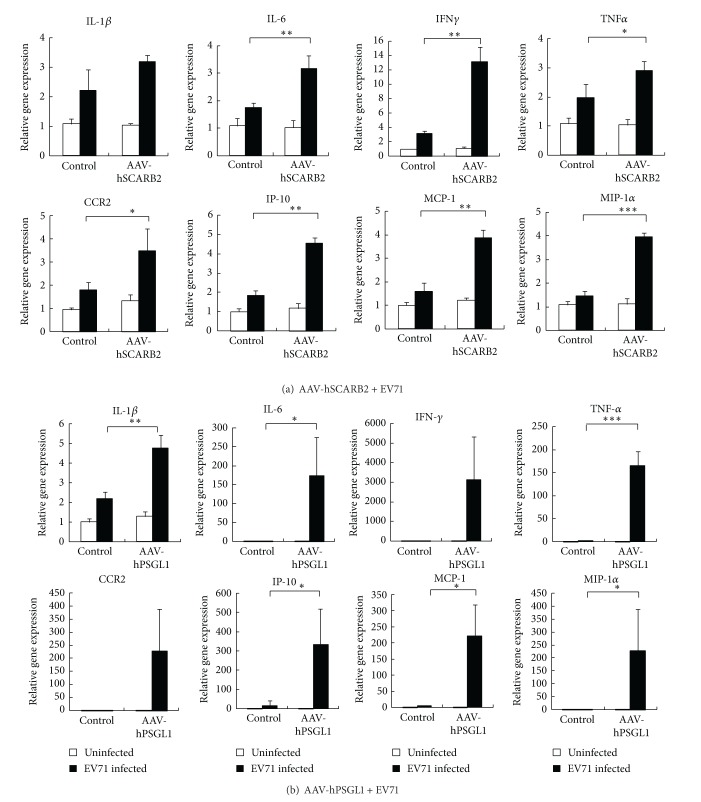
Upregulation of cytokines or chemokines in brain tissue in rAAV-hSCARB2- and rAAV-hPSGL1-transduced mice. Adult ICR mice were i.v. injected and orally administered 100 *μ*L of 5 × 10^12^ vg/mL of rAAV-hSCARB2 (a) or rAAV-hPSGL1 (b) and then infected with EV71 (1 × 10^7^ pfu in 100 *μ*L) three weeks later via i.p. injection. Seventy-two hours after EV71 infection, RNA was extracted from the brain tissue and analyzed by real-time RT-PCR to determine the expression of cytokines (IL-1*β*, IL-6, IFN*γ*, and TNF*α*), chemokines (CCR2, IP-10, MCP-1, and MIP-1*α*), and GAPDH. RNA was also extracted from untreated mice as control. The related gene expression was calculated using the comparative method for the relative quantity normalized to GAPDH gene expression. *P < 0.05, **P < 0.01, and ***P < 0.001 (*t*-test). All of the samples were collected from three individual animals (*N* = 3).

Induction of proinflammatory cytokines has been reported correlated with the pathogenesis of EV71 infection [9, 13]. Therefore, we measured the induction of proinflammatory cytokines in the EV71 receptor-expressing mice infected with EV71. The data in Figure 5(a) showed that IL-6, TNF*α*, and IFN*γ* significantly increased 2–6 folds in the brain tissue of rAAV-hSCARB2-transduced mice after EV71 4643 infection. However, in the intestinal tissue, only IFN*γ* could be detected at an increasing rate in the rAAV-hSCARB2-transduced mice (Supplementary Figure S2). To further investigate the expression levels of chemokines in the brain tissue after EV71 infection, we observed that the expression of CCR2, IP-10, MCP-1, and MIP-1*α* increased 2-fold after EV71 infection. Accordingly, the rAAV-hPSGL1-transduced mice that were infected with EV71 also showed higher levels of cytokines and chemokines than the control mice (Figure 5(b)). These results demonstrated that proinflammatory cytokines and chemokines were induced in both rAAV-hSCARB2- and rAAV-hPSGL1-transduced mice after EV71 infection. This method is a potential model for the development of vaccines or drugs for EV71.

## 4. Discussion

Previous evidence has shown that mice that are more than 4-week old are generally not susceptible to EV71 [19, 21, 36, 37]. Thus, an adult animal model is required to better understand the pathogenesis of EV71 infection and to help develop vaccines and drugs. Here, we demonstrated that by using an adeno-associated virus (AAV) delivery system, we were able to introduce EV71 receptors into adult mice, increasing their susceptibility to EV71 infection. Although hSCARB2 or hPSGL1 expression in mice increases EV71 infection, gene expression levels are different in various organs. The majority of the hSCARB2 is expressed in the lungs and liver, and in contrast, a large amount of hPSGL1 was expressed in the brain and kidney (Figure 3(a)). This difference may be caused by the basic character of these two proteins. Since SCARB2 has already been found to be capable of expressing in a variety of cell types and tissues, and PSGL-1 is mainly found to express on the cell-surface of leukocytes, so it is not surprised to observe the general gene expression level of hSCARB2 to be higher than hPSGL1 (Figure 3(a)). However, we cannot exclude the possibility that this difference may be just because of the different gene expression efficiencies between these two recombinant AAVs. After EV71 infection, virus replication titers were comparable in the brain and intestines in both hSCARB2- and hPSGL1-expressing mice (Figure 4). These results indicate that hPSGL1, as a receptor of EV71, is able to support viral replication comparable to those obtained from hSCARB2 in the current animal model studies. Previous studies have shown that the tissue tropism and pathogenesis of EV71 infection were determined and/or regulated by combinations of several factors [38]. Although previous reports had demonstrated that EV71 could infect hSCARB2-expressing cells more efficiently than the PSGL1-expressing cells [39], the host factors might be important and affect EV71 replication in different organs of the AAV-delivered mice. The hSCARB2 transgenic mice were reported to be more susceptible to EV71 infection than the hPSGL1 transgenic mice and resulted in significant neurological syndrome [24, 25, 40]; we did not observed these differences in this study.

Because severe EV71 infections associated with brainstem encephalitis were correlated with the local production of proinflammatory cytokines, the expression of EV71 receptors in the brain may induce the production of proinflammatory cytokines after EV71 infection. Our studies showed that cytokines induced by EV71 infection were significantly increased in mice that received both rAAV-hPSGL1 and rAAV-hSCARB2 (Figure 5(a)). These results were consistent with previous studies in EV71-infected patients who demonstrated massive IL-1*β*, IL-6, IFN*γ*, and TNFα secretion in the brainstem [8, 9, 15]. We also analyzed several chemokines expressed in the brain after EV71 infection. Mice expressing the EV71 receptors, hPSGL1, or hSCARB2, displayed a significant increase in the expression of IP-10, MCP-1, and MIP-1*α* compared with the control mice (Figure 5(b)). These results are consistent with other studies that indicated that these chemokines are related to the progress of EV71-induced fatal neurological symptoms and acute respiratory failure [41]. The induction of cytokines or chemokines in hPSGL1-expressing mice is dramatically higher compared with hSCARB2-expressing mice and may reflect the high expression level of hPSGL1 in the mouse brain (Figure 3(a)). In addition, PSGL-1 has been shown, that is, primarily expressed in leukocytes [42]. We speculated that hPSGL1 would express on the leukocytes cell surface of AAV-transduced mice and upon EV71 infection large amount of chemokines or cytokines are induced.

However, no typical syndromes, such as ataxia and paralysis, were observed after EV71 infection in our mouse model, which may be because a single EV71 receptor is not sufficient to induce pathogenesis in adult mice or because the immune system in adult mice can overcome the virus-induced disorders. The other potential weakness of this study is the use of naïve ICR mice as control mice. Although previous studies have already proved AAV vector can stably express target genes* in vivo* without triggering immune responses to the vectors itself, we still could not exclude the possibility of potential nonspecific immunological effect induced by AAV.

In combination, the delivery of EV71 receptors by recombinant AAV increased EV71 replication in the brain and intestines and may be used to study the efficacy of EV71 vaccines or anti-EV71 drugs. Our recent study has found that 7-day-old TLR9 knockout mice are more sensitive to the EV71 infection compare to the wild type mice (unpublished data). It is of interest to know whether the gene transfer EV71 receptors into adult TLR9 knockout mice could increase the susceptibility to EV71 infection. The positive results will support the potential roles of TLR9 in both EV71 infection and pathogenesis. Furthermore, the recombinant AAV delivery system provides more flexibility and advantages and allows the introduction of different recombinant AAV constructs into different strains of mice for investigating the roles of innate receptors during EV71 infection.

## Supplementary Material

Supplementary table 1. provides the information of primers used in this study for all of the real-time PCR analysis.
Supplementary figure S1. is the results of using immune- fluorescence assay to confirm the expression of EV71 protein in L929 cells which were transduced with AAV-hSCARB2 and AAV-hPSGL1 and followed with EV71 infection.
Supplementary figure S2. provides the results of cytokines and chemokines expression in intestinal tissues from AAV-hSCARB2- and AAV-hPSGL1-transduced mice followed with EV71 infection.
