# Supplementary material for: Delivery of Human EV71 Receptors by Adeno-Associated Virus Increases EV71 Infection-Induced Local Inflammation in Adult Mice
Source: Biomed Res Int. 2014 Aug 27;2014:878139. doi: 10.1155/2014/878139 (PMC4163470; doi:10.1155/2014/878139)
Supplement: Supplementary file 1 — Supplementary table 1. provides the information of primers used in this study for all of the real-time PCR analysis. Supplementary figure S1. is the results of using immune- fluorescence assay to confirm the expression of EV71 protein in L929 cells which were transduced with AAV-hSCARB2 and AAV-hPSGL1 and followed with EV71 infection. Supplementary figure S2. provides the results of cytokines and chemokines expression in intestinal tissues from AAV-hSCARB2- and AAV-hPSGL1-transduced mice followed with EV71 infection. [file 878139.f1.pdf]

**Supplemental table 1.** The UPL number and primer sequences for mRNA analysis by real-time PCR. Sequences designed for the detection of indicated gene products by real-time PCR are presented

| Gene name | UPL no. | primers |
|---|---|---|
| EV71 VP1 | 2 | Forward: 5′-atgacgggtatcccacattc-3′ |
| | | Reverse: 5′-catgccccgtattcaaggt-3′ |
| IL-1β | 78 | Forward: 5′-tgtaatgaaagacggcacacc-3′ |
| | | Reverse: 5′-tcttctttgggtattgcttgg-3′ |
| GAPDH | 9 | Forward: 5′-gagccaacgggtcatcatct-3′ |
| | | Reverse: 5′-gaggggccatccacagtctt-3′ |
| hPSGL-1 | 10 | Forward: 5′-agaatccaggccgtgaaaa-3′ |
| | | Reverse: 5′-ggtgcttggcacctctgt-3′ |
| hSCARB2 | 48 | Forward: 5′-gaaacgggagacattagaacca-3′ |
| | | Reverse: 5′-cgccgtctctttatcaatgtg-3′ |
| TNF-α | 25 | Forward: 5′-ctgtagcccacgtcgtagc-3′ |
| | | Reverse: 5′-ttgagatccatgccgttg-3′ |
| IFN-γ | 21 | Forward: 5′-atctggaggaactggcaaaa-3′ |
| | | Reverse: 5′-ttcaagacttcaaagagtctgagg-3′ |
| IL-6 | 6 | Forward: 5′-gctaccaaactggatataatcagga-3′ |
| | | Reverse: 5′-ccaggtagctatggtactccagaa-3′ |
| IP-10 | 3 | Forward: 5′-gctgccgtcatttctgc-3′ |
| | | Reverse: 5′-tctcactggcccgtcatc-3′ |
| MCP-1 | 62 | Forward: 5′-catccacgtgttggctca-3′ |
| | | Reverse: 5′-gatcatcttgctggtgaatgagt-3′ |
| MIP-1α | 40 | Forward: 5′-caagtcttctcagcgccata-3′ |
| | | Reverse: 5′-ggaatcttccggctgtagg-3′ |
| CCR2 | 27 | Forward: 5′-acctgtaaatgccatgcaagt-3′ |
| | | Reverse: 5′-tgtcttccatttcctttgatttg-3′ |

**Supplementary figure S1**

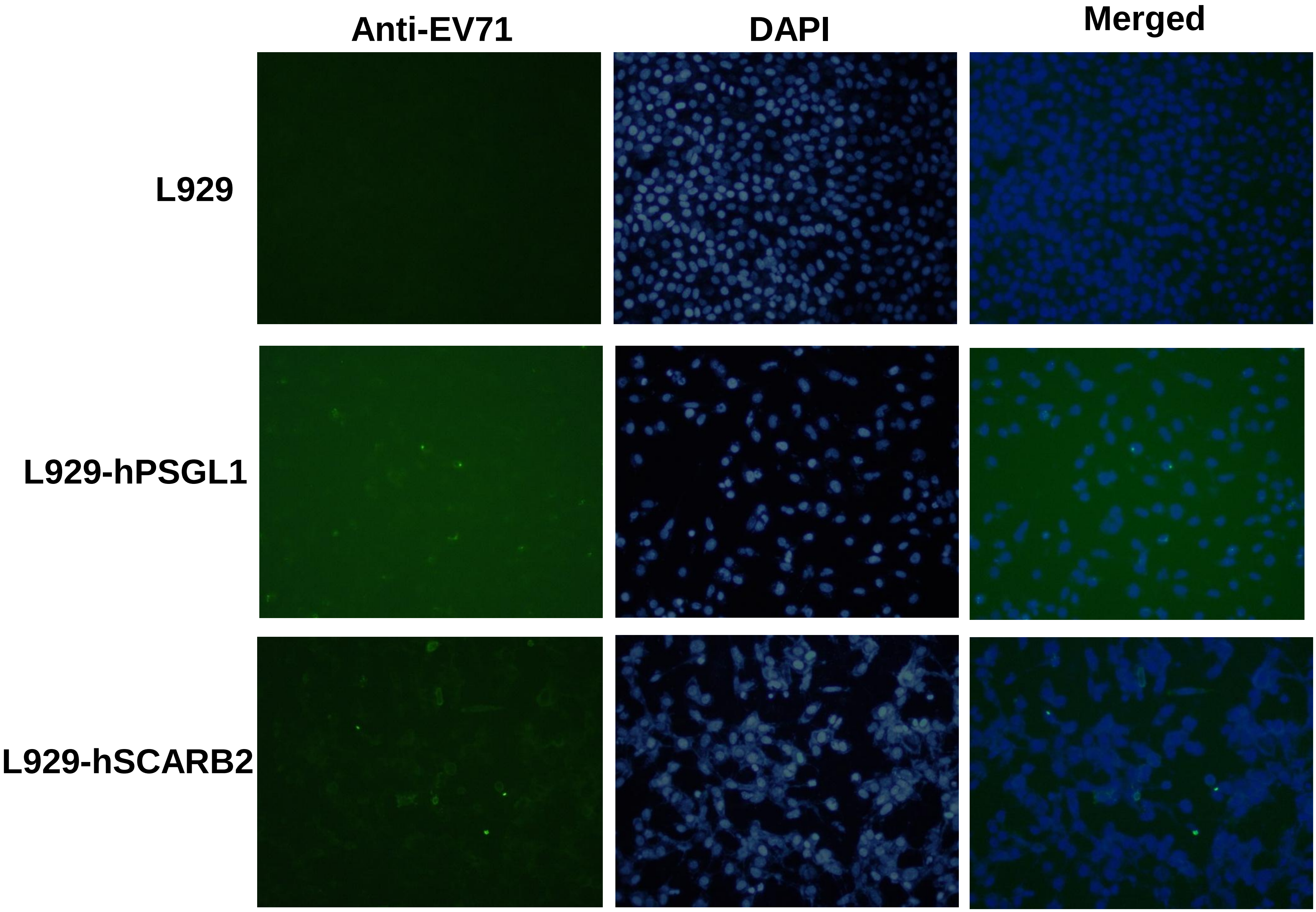

**Supplementary figure S1.**

**EV71 was detected in hSCARB2- and hPSGL1-expressed L929 cells, but not in the control cells.** L929 cells were seeded into a 24-well plate ($5x10^5$ per well) one day prior experiment, and AAV-hPSGL1 and -hSCARB2 were transfected into L929 cells by using PolyJet™ In Vitro DNA Transfection Reagent (SignaGen® Laboratories). Transfected and non-transfected L929 cells were then infected with EV71 at MOI 10. After 24hr, EV71-infected cells were washed with PBS and fixed in 3.7% paraformaldehyde for 10 min at room temperature. The cells were incubated for 30 min at room temperature with a 1:1,000 dilution of monoclonal antibody (MAb) against EV71 (MAb979; Chemicon International, Temecula, CA). A 1:200 dilution of FITC-conjugated secondary antibodies (Jackson ImmunoResearch Laboratory, Baltimore, PA) was added and counterstained with DAPI (Sigma, St. Louis, MO). All of the cells were washed three times with sterile PBS and monitored by a fluorescence microscope.

# Supplementary figure S2

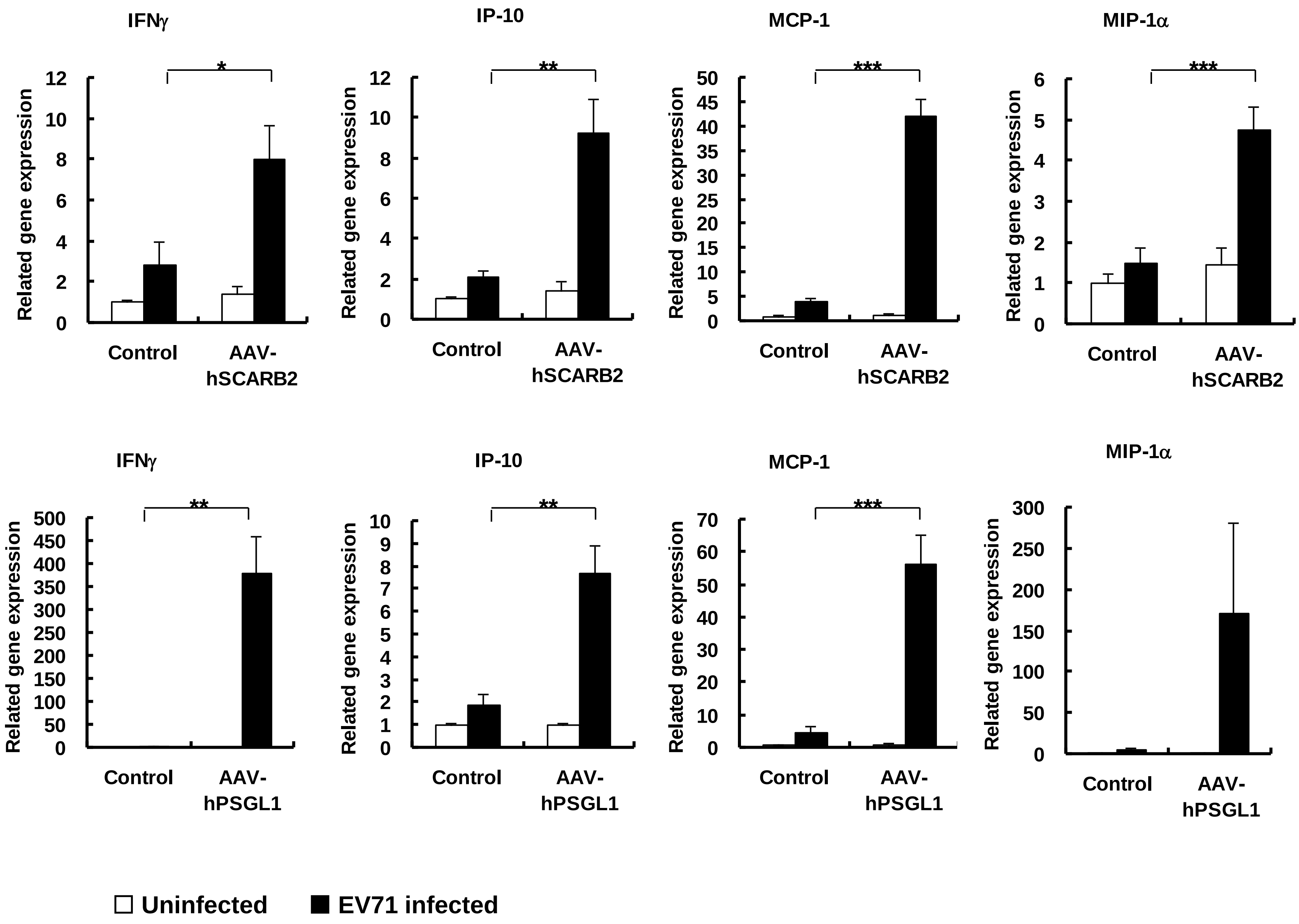

**Supplementary figure S2.**

**Up-regulation of cytokines or chemokines in intestinal tissue in rAAV-hSCARB2- and rAAV-hPSGL1-transduced mice.** Adult ICR mice were i.v. injected and orally administered 5 x $10^{11}$ pfu of rAAV-hSCARB2 or rAAV-hPSGL1 and then infected with EV71 (1x$10^{7}$ pfu in 100 μL) three weeks later via i.p. injection. Seventy-two hours after EV71 infection, RNA was extracted from the intestines and analyzed by real-time RT-PCR for the expression of cytokines (IFNγ), chemokines (IP-10, MCP-1, and MIP-1α), and GAPDH. RNA was also extracted and analyzed from untreated mice as control. The related gene expression was calculated using the comparative method for the relative quantity normalized to GAPDH gene expression. *: $p<0.05$, **: $p<0.01$, ***: $p<0.001$ (*t* test).
